# Chemotherapy-induced Sinusoidal Injury (CSI) score: a novel histologic assessment of chemotherapy-related hepatic sinusoidal injury in patients with colorectal liver metastasis

**DOI:** 10.1186/s12885-016-2998-2

**Published:** 2017-01-07

**Authors:** Heather L. Stevenson, Mariana M. Prats, Eizaburo Sasatomi

**Affiliations:** 1 Department of Pathology, The University of Texas Medical Branch, John Sealy Annex Building - Room 2.148, 301 University Blvd., Galveston, TX 77555-0588 USA; 2 Department of Pathology, The University of Texas Medical Branch, John Sealy Annex Building, 301 University Blvd., Galveston, TX 77555-0588 USA; 3 Department of Pathology and Laboratory Medicine, The University of North Carolina at Chapel Hill Women’s & Children’s Hospitals, Room 30212, 101 Manning Drive, Chapel Hill, NC 27514 USA

**Keywords:** Colorectal liver metastasis, Sinusoidal obstruction syndrome, Oxaliplatin, FOLFOX, Impaired liver function

## Abstract

**Background:**

Preoperative neoadjuvant therapy for colorectal liver metastases (CRLM) is increasing in use and can lead to chemotherapy-induced damage to sinusoidal integrity, namely sinusoidal obstruction syndrome (SOS). SOS has been associated with an increased need for intraoperative blood transfusions, increased length of hospitalization post-surgery, decreased tumor response, and a shorter overall survival after resection due to liver insufficiency. It is critical for clinicians and pathologists to be aware of this type of liver injury, and for pathologists to include the status of the background, non-neoplastic liver parenchyma in their pathology reports. In this study, expression of CD34 by sinusoidal endothelial cells (SECs), increased expression of smooth muscle actin (SMA) by hepatic stellate cells (HSCs), and aberrant expression of glutamine synthetase (GS) by noncentrizonal hepatocytes were semiquantitatively evaluated in liver resection or biopsy specimens from patients with CRLM to determine their diagnostic value for assessing chemotherapy-induced sinusoidal injury (CSI).

**Methods:**

The expression of each marker was compared among 22 patients with CRLM with histologically evident SOS (SOS+) and 8 patients with CRLM who had not undergone chemotherapy. Each case was given a histologic grade using the sinusoidal obstruction syndrome index score (SOS-I) to assess the likelihood of SOS. Cases were also given an immunohistochemical grade using the total CSI score calculated as the sum of CD34, SMA, and GS scores.

**Results:**

Abnormal staining patterns for CD34 and SMA were significantly more frequent and extensive in SOS+ cases than in the controls (81.8% vs. 25%, *P* < 0.01; 72.7% vs. 25%, *P* = 0.03). Aberrant GS expression in midzonal and periportal hepatocytes was only observed in SOS+ cases (31.8% vs. 0%), but this difference did not reach statistical significance. The CSI score was significantly higher in the SOS+ cases when compared to controls (*P* < 0.01), and was associated with a higher SOS histologic grade (*P* = 0.02).

**Conclusions:**

The CSI score, calculated using an immunohistochemical panel consisting of CD34, SMA, and GS, may serve as an objective marker of chemotherapy-induced sinusoidal injury and could help diagnose this peculiar form of liver injury.

## Background

The liver is the most frequent site of colorectal cancer metastases. Approximately 15 to 25% of patients present with synchronous, and 25 to 30% develop metachronous liver metastasis [1]. Curative metastasectomy is possible in the setting of liver-limited metastases; however, only 15 to 20% of patients are considered candidates for hepatic resection at the time of presentation. Systemic chemotherapy in the preoperative setting is increasingly used to improve the potential benefit of surgery and to downstage initially unresectable disease, allowing for potentially curative surgery [2, 3]. The latter strategy, referred to as “conversion chemotherapy” or “downsizing therapy,” is a major reason for the yearly increase in the number of liver resections for colorectal liver metastases (CRLM). The most commonly used CRLM chemotherapy regimens include oxaliplatin (OX) plus 5-fluorouracil (5-FU) and leucovorin (FOLFOX) and irinotecan plus 5-FU and leucovorin (FOLFIRI). As chemotherapy is often administered prior to hepatic resection, adverse effects on the background liver parenchyma of CRLM patients are increasingly recognized [4, 5]. Recent meta-analysis studies have demonstrated that the nature of preoperative chemotherapy liver injury is a regimen-specific phenomenon [6]. For example, OX-based regimens are associated with sinusoidal injury, whereas irinotecan-based regimens are associated with steatohepatitis. In the context of liver surgery, chemotherapy-induced liver injury could increase the risks of intra- and postoperative complications and postoperative liver insufficiency [7].

Sinusoidal obstruction syndrome (SOS), previously termed veno-occlusive disease (VOD), is considered the result of severe toxic injury affecting hepatic sinusoidal endothelial cells (SECs). SOS has been associated with the use of OX-based systemic chemotherapy, cytoreductive therapy prior to hematopoietic stem cell transplantation, hepatic irradiation, pyrrolizidine alkaloid-containing herbal remedies, liver transplantation, and a rare autosomal recessive disorder of the liver and immune system (hepatic veno-occlusive disease with immunodeficiency) [8]. Macroscopically, the affected liver typically has a characteristic bluish-red marbled appearance and therefore, has been called “blue liver syndrome”. Histologically, SOS is characterized by distinct areas of dilated sinusoids with congestion, which may be associated with liver cell plate atrophy. In severe cases, it can also be associated with perisinusoidal fibrosis, nodular regenerative hyperplasia (NRH), obstruction of centrilobular veins, and peliotic change [9]. The reported incidence of OX-induced SOS varies between 8.3 and 54% [9, 10]. Patients with OX-induced SOS in the setting of CRLM have significantly impaired functional hepatic reserve and are predisposed to increased blood transfusions and higher morbidity after the hepatic resection [7]. Recent studies have demonstrated that SOS induced by OX-based chemotherapy might diminish the response to chemotherapy in patients with CRLM [11, 12]. SOS may also compromise liver regeneration in patients undergoing hepatectomy [13]. Therefore, both pathologists and clinicians should be aware of this syndrome as it has a relatively high prevalence and may affect patient outcomes.

“Sinusoidal capillarization” is the altered phenotype of SECs characterized by increased CD34 expression, basement membrane formation, and decreased fenestrae numbers, and is typically seen at the periphery of cirrhotic nodules. In cases of OX-induced liver injury, increased CD34 expression by SECs has been associated with the severity of sinusoidal injury and deterioration of hepatic functional reserve [14, 15]. It is well known that hepatic stellate cells (HSCs) transform from a quiescent vitamin A-rich type to a myofibroblastic fibrogenic phenotype that can produce excessive amounts of extracellular matrix, leading to perisinusoidal fibrosis [16]. While centrilobular perisinusoidal/venular fibrosis is often reported in cases with OX-induced liver injury, the expression status of SMA by HSCs has only been sporadically described in this clinical setting [17, 18]. In the normal liver, GS is expressed exclusively in a narrow rim of pericentral hepatocytes; however, the area of GS-expressing hepatocytes has recently been shown to be significantly expanded in several pathologic conditions including focal nodular hyperplasia (FNH), liver cirrhosis of various etiologies, and idiopathic portal hypertension [19–22]. Because these conditions are associated with altered intrahepatic blood flow caused by shunt vessels or the presence of aberrant vasculature, impaired sinusoidal microcirculation in SOS may also lead to altered GS expression in the hepatic lobules.

The primary objective of this study was to assess the value of immunohistochemical labeling for CD34, SMA, and GS in the diagnosis of SOS after chemotherapy for CRLM and to develop a scoring system that could be easily implemented by pathologists. Each case was given a histologic grade using the sinusoidal obstruction syndrome index score (SOS-I) to assess the likelihood of SOS. Cases were also given an immunohistochemical grade using the total chemotherapy-induced sinusoidal injury (CSI) score calculated as the sum of CD34, SMA, and GS scores. The relationship between the CSI score and histologic disease severity was also assessed.

## Methods

### Patient and tissue selection

Following institutional review board approval, this study was conducted in the Department of Pathology at the University of Pittsburgh Medical Center (UPMC), Presbyterian Hospital. Between January 2012 and December 2013, 121 patients (74 males and 47 females, aged 28 to 91 years, median: 60 years) underwent liver resection for either synchronous or metachronous CRLM or liver biopsy to evaluate the background liver parenchyma. Before the procedure, 72 patients were treated with OX-based chemotherapy, 16 patients were initially treated with OX-based chemotherapy and later converted to irinotecan-based chemotherapy, 9 were treated with irinotecan-based chemotherapy, and 13 were treated with either 5-FU or oral capecitabine alone. Eleven subjects received no chemotherapy. Among these patients, routine histology slides were available in 107 cases. For these cases, hematoxylin & eosin (H&E)-stained non-neoplastic liver parenchyma slides were blindly and independently evaluated by two hepatopathologists (H.L.S. and E.S.). Three exclusion criteria were applied to minimize confounding effects of other possible significant liver injuries and secondary changes resulting from surgery and/or a possible tumor mass effect. These included: 1) the presence of moderate to severe steatosis (steatosis involving greater than 33% of hepatocytes) 2) cases in which the only non-neoplastic liver parenchyma was within 1.5 cm of the cauterized margin or tumor nodules, and 3) cases with well-developed bridging fibrosis or nodule formation. As a result, 40 cases with available slides not meeting the above criteria were excluded from the remainder of the study. For the remaining 67 cases, the likelihood of SOS was assessed based on: 1) the degree of sinusoidal injury, 2) the presence or absence of NRH, and 3) the presence or absence of partial or total venous obstruction as shown in Table 1. The SOS-I was then calculated by summing the sinusoidal injury, NRH, and venous occlusion scores in each case similar to that described previously by Rubbia-Brandt et al. [9]. For those with an SOS-I ≥2 and 0/1, the cases were classified as SOS+ and SOS–, respectively. Of the 67 cases evaluated, all 11 control cases were SOS–. Among the cases treated with chemotherapy, 31 (55.4%) cases were SOS– and 25 (44.6%) cases were SOS+. Of these cases, tissue blocks were available for 8 controls and 22 SOS+ cases. Masson’s trichrome and immunohistochemical stains for CD34, SMA, and GS were performed in these cases. The trichrome stain was blindly evaluated by two hepatopathologists (H.L.S. and E.S.) and scored as 0 or 1, as shown Table 2.

**Table 1:** Histologic grading of sinusoidal obstruction syndrome

Sinusoidal Injury (SI)
0 - No sinusoidal dilatation or congestion
1 - patchy/confluent areas of sinusoidal dilatation/congestion without hepatocyte atrophy
2 - patchy/confluent areas of sinusoidal dilatation/congestion with associated mild hepatocyte atrophy
3 - patchy/confluent areas of sinusoidal dilatation/congestion with associated moderate or severe hepatocyte atrophy
Nodular Regenerative Hyperplasia (NRH)
0 - absent
1 - present
Venous Obstruction (VO)
0 - absent
1 - present, partial obstruction of central venule(s)
2 - present, total occlusion of central venule(s)

Adapted from Rubbia-Brandt et al. [9]

**Table 2:** Grading of trichrome stain

0 - no perisinusoidal fibrosis
1 - perisinusoidal fibrosis in the areas of sinusoidal dilatation/hepatocyte atrophy

### Immunohistochemistry

Routinely formalin-fixed and paraffin-embedded liver samples were immunostained with indirect immunohistochemistry using monoclonal antibodies to CD34, SMA, and GS, followed by the appropriate secondary antibodies. The details of the primary antibodies used, their dilutions, and the procedure for antigen retrieval are summarized in Table 3. The reactions were revealed with 3-amino-9-ethylcarbazol (ScyTek Laboratories, West Logan, UT, USA) as a chromogen and then lightly counterstained with modified Gill’s hematoxylin (Ventana Medical Systems, Tucson, AZ, USA). The immunohistochemical stains for CD34, SMA, and GS were blindly evaluated by one of the authors (E.S.) in a semiquantitative manner as shown in Table 4. Briefly, each stain was evaluated and given a staining intensity score of 0 to 3; the results of which were summed to give the CSI score.

**Table 3:** Antibodies used in the study and their specifications

Antibody	Cat number/clone	Source	Dilution	Antigen retrieval
CD34	M7165/clone QBEnd-10	DAKO (Carpinteria, CA, USA)	1:25	Steam slides in preheated Target Retrieval Solution, pH 9 (Carpinteria, CA, USA) for 30 min
SMA	M0851/clone 1A4	DAKO (Carpinteria, CA, USA)	1:50	None
GS	MAB302/clone GS-6	EMD Millipore (Billerica, MA, USA)	1:2000	Steam slides in pre-heated Target Retrieval Solution, Citrate pH 6 (Carpinteria, CA, USA) for 60 min

**Table 4:** Grading of CD34, smooth muscle actin and glutamine synthetase staining

CD34	
0 - no periportal sinusoidal staining or focal, (non-circumferential) periportal sinusoidal staining	
1 - circumferential periportal sinusoidal staining present	
2 - bridging (zone 1 to zone 1) sinusoidal staining present	
3 - 2 and midzonal to centrizonal (zone 2 to zone 3) staining	
Smooth Muscle Actin	
0 - no apparent staining or rare isolated positive cells in the perisinusoidal space	
1 - many positive cells without linear/continuous pattern of perisinusoidal staining	
2 - focal linear/continuous pattern of perisinusoidal staining	
3 - multifocal linear/continuous perisinusoidal staining	
Glutasmine Synthetase	
0 - normal perivenular staining pattern	
1 - focal midzonal (zone 2) staining present	
2 - multifocal midzonal (zone 2) staining present	
3 - unequivocal confluent midzonal to periportal staining, if any	

### Statistics

Comparisons between the two groups were carried out with Mann–Whitney U test or Fisher’s exact test using StatPlus:mac Pro (Version 6.1, AnalystSoft Inc., Walnut, CA). Differences were considered significant at *P* < 0.05.

## Results

### Histological evaluation

**Fig. 1:**
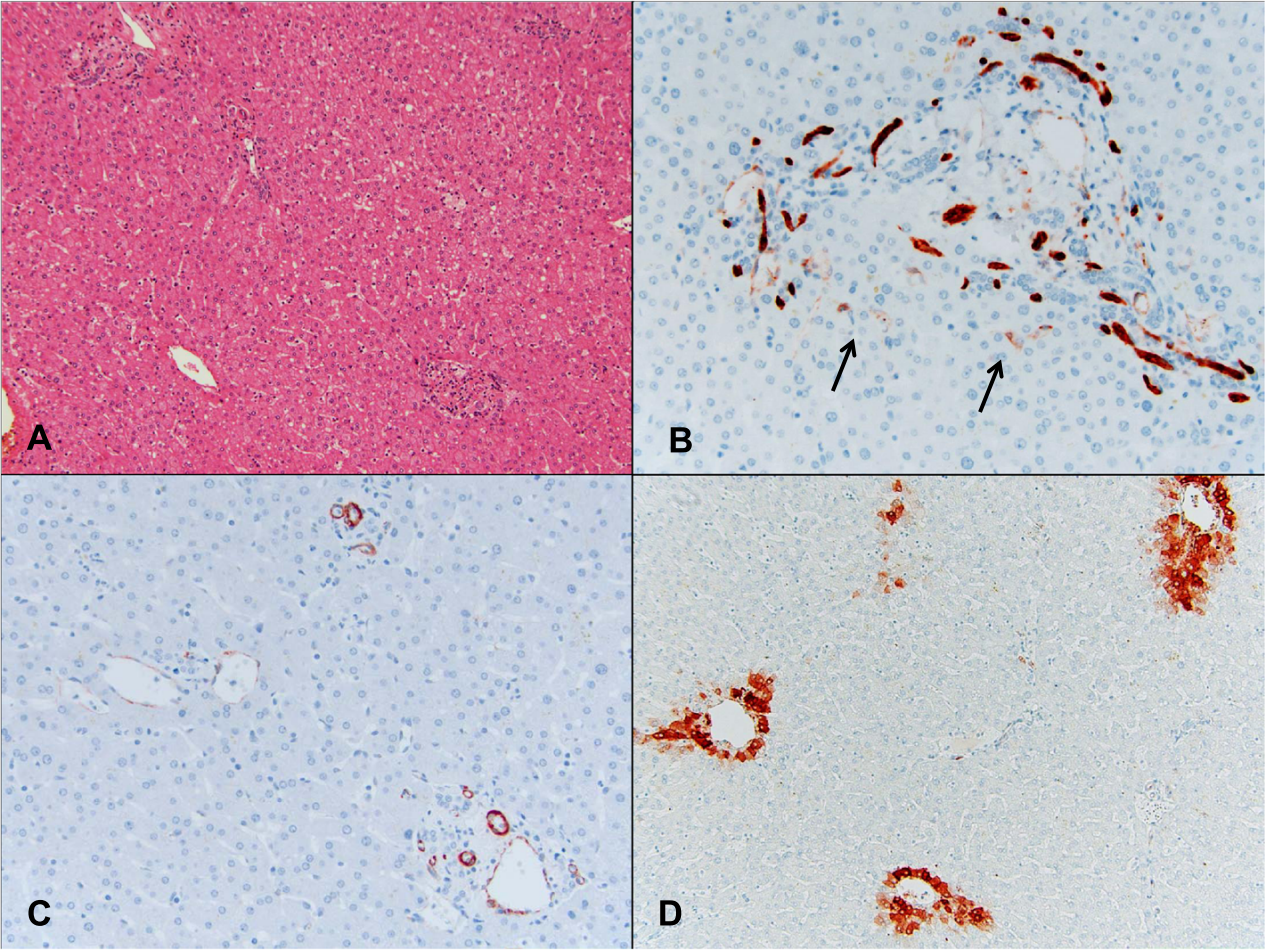
An example of a control group (case 29 in Table 5) in which a patient with CLRM did not receive chemotherapy prior to resection. **a** This case showed no significant sinusoidal dilatation, congestion, or parenchymal nodularity (H&E, ×40). **b** An immunohistochemical stain for CD34 showed the normal staining pattern with only focal weak sinusoidal staining around the portal tracts (arrows, ×200). **c** Positive immunoreactivity for smooth muscle actin (SMA) was seen in the portal blood vessels, bile ducts, and central venules. No SMA-positive stellate cells were present in the lobules (×200). **d** An immunohistochemical stain for GS revealed a normal perivenular staining pattern (×100)

**Fig. 2:**
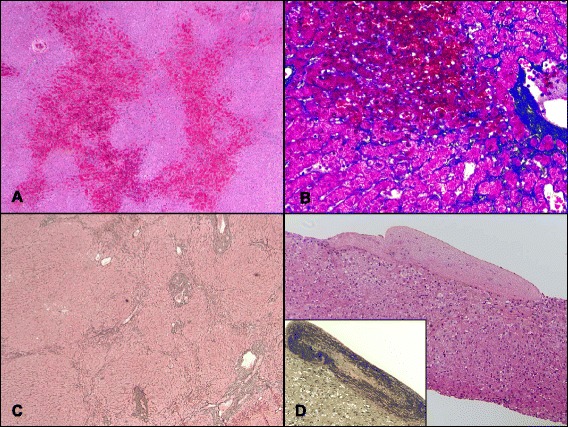
Common histologic features of SOS. **a** Shown are changes typical of sinusoidal obstruction (Case 12). Bridging bands of congestion were observed connecting centrizonal areas (H&E, ×40). **b** In the same case, a trichrome stain highlights prominent fibrosis extending along centrilobular sinusoids and also shows atrophic hepatocyte trabeculae (trichrome stain, ×200). **c** Nodular regenerative hyperplasia. In some cases (e.g., Case 6), the hepatic parenchyma showed diffuse transformation into small nodules with little to no fibrosis (reticulin stain, ×40). **d** Venous occlusion. Case 14 showed complete fibrous obliteration of the central veins (H&E, ×100), which was further confirmed with Verhoeff–Van Gieson staining (inset, ×100)

The histologic grade and chemotherapy details in each of these 30 cases are summarized in Table 5. The histologic findings of representative cases are shown in Fig. 1a (control) and Fig. 2a-d (SOS+ cases). Of the 22 SOS+ cases, 17 were treated with OX-based chemotherapy, 4 were initially treated with OX-based chemotherapy and later converted to irinotecan-based chemotherapy, and 1 was principally treated with irinotecan-based chemotherapy. The number of treatment cycles with OX-based chemotherapy was available in 15 cases, and the average was 5.5. In our series, unequivocal venous occlusion was found in only one SOS+ case treated with a FOLFOX regimen (4.5%) (Table 5, Case 14; see also Fig. 2d), and most cases were categorized as SOS+ based on a combination of sinusoidal injury and NRH scores (63.6%) or sinusoidal injury score alone (31.8%). The SOS-I score of the control cases was less than 2 in all cases, and the majority had an SOS-I of score 1 (75%, 6/8), mostly due to sinusoidal dilatation and congestion without significant hepatocyte atrophy. The trichrome stain highlighted perisinusoidal fibrosis in 59% (13/22) of SOS+ cases and 37.5% (3/8) of control cases. There was no statistical difference in the trichrome staining results between the SOS+ and control groups (*P* = 0.42).

**Table 5:** Expression of CD34, smooth muscle actin and glutamine synthetase in patients with chemotherapy-induced SOS

Case		Histologic Grade				Trichrome	Immunohistochemistry Grade				
No.	Age^a^	SI	NRH	VO	SOS-I		CD34	SMA	GS	CSI score	Chemotherapy (cycle #)
1	70–74	3	1	0	4	1	3	3	0	6	capecitabine and oxaliplatin (2)
2	65–69	3	1	0	4	1	3	3	0	6	FOLFOX (4)
3	65–69	3	1	0	4	0	3	3	0	6	capecitabin, oxaliplatin (2) and cetuximab
4	55–59	3	1	0	4	0	3	1	3	7	FOLFOX (*) and bevacizumab
5	65–69	3	1	0	4	1	3	2	3	8	FOLFOX (*)
6	60–64	3	1	0	4	1	3	3	3	9	FOLFOX (6)
7	70–74	3	1	0	4	0	2	3	0	5	FOLFOX and bevacizumab (6)
8	50–54	2	1	0	3	1	0	0	0	0	FOLFOX (6)
9	70–74	2	1	0	3	1	2	0	0	2	FOLFOX (*)
10	65–69	2	1	0	3	0	2	3	0	5	FOLFOX (*) and bevacizumab
11	65–69	3	0	0	3	1	3	3	2	8	FOLFOX (4)
12	55–59	3	0	0	3	1	3	3	1	7	FOLFOX (3) and bevacizumab
13	75–79	2	1	0	3	0	3	0	0	3	FOLFOX (*)
14	70–74	0	1	2	3	1	0	3	3	6	FOLFOX (12)
15	30–34	1	1	0	2	0	2	3	0	5	FOLFOX (8), converted to FOLFIRI and bevacizumab
16	50–54	2	0	0	2	1	2	3	0	5	FOLFOX (6) and cetuximab
17	50–54	2	0	0	2	0	0	0	0	0	FOLFOX (8), converted to FOLFIRI, bevacizumab and cetuximab
18	55–59	2	0	0	2	0	0	3	0	3	FOLFOX (4) and bevacizumab
19	45–49	2	0	0	2	1	1	3	3	7	FOLFOX (5) and cetuximab converted to FOLFIRI
20	55–59	1	1	0	2	0	1	0	0	1	FOLFOX (6)
21	55–59	2	0	0	2	1	2	3	0	5	FOLFOX (*), converted to FOLFIRI, avastin and cetuximab
22	50–54	1	1	0	2	1	2	0	0	2	FOLFIRI and cetuximab
23	55–59	1	0	0	1	1	0	2	0	2	none
24	60–64	1	0	0	1	0	1	0	0	1	none
25	50–54	0	1	0	1	1	2	0	0	2	none
26	70–74	1	0	0	1	0	0	0	0	0	none
27	60–64	1	0	0	1	1	0	3	0	3	none
28	55–59	1	0	0	1	0	0	0	0	0	none
29	85–89	0	0	0	0	0	0	0	0	0	none
30	75–79	0	0	0	0	0	0	0	0	0	none

^a^Ages were replaced by age ranges to maintain partient confidentiality

*SOS* sinusoidal obstruction syndrome, *SI* sinusoidal injury, *NRH* nodular regenerative hyperplasia, *VO* venous obstruction, *SOS-I* SOS index, *SMA* smooth muscle actin, *GS* glutamine synthetase, *CSI score* chemotherapy-induced sinusoidal injury score, *OX* oxaliplatin, *unknown cycle

### Staining profile for CD34, SMA, and GS

**Fig. 3:**
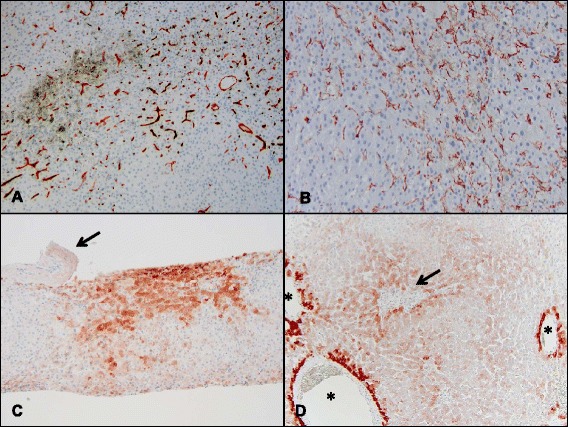
Aberrant expression of CD34, SMA, and GS in cases with sinusoidal obstruction syndrome were used to calculate the CSI score. **a** In case 12, the immunohistochemistry for CD34 showed confluent sinusoidal staining extending from the periportal to midzonal and centrizonal areas (×100). This is an example of a immunohistochemistry grade 3 for CD34. **b** Immunohistochemical stain for SMA in the same case showed confluent subsinusoidal staining in multiple areas (×200), which is also an example of a grade 3 staining intensity. **c** Immunohistochemistry conducted on case 14 showed GS expression adjacent to an obliterated sublobular vein (arrow) that was expanded (×100) compared to the normal perivenular GS staining pattern and was also give an immunohistochemistry score of 3. **d** In Case 19, aberrant confluent GS expression is seen in midzonal and periportal areas; another example of a grade 3 pattern of staining (asterisks, central veins; arrow, portal tract; ×100)

The immunohistochemical results for CD34, SMA, and GS are summarized in Table 5. The normal staining patterns of CD34, SMA, and GS in a control case are shown in Fig. 1, and the abnormal staining patterns of CD34, SMA, and GS observed in representative SOS+ cases are shown in Fig. 3. Aberrant CD34 expression in sinusoidal lining cells was significantly more frequent in SOS+ cases (81.8%, 18/22) than in controls 25% (2/8) (*P* < 0.01), and the CD34 score of SOS+ cases was also significantly higher than that of the controls (*P* < 0.01). As reflected in the scoring system (see Table 4), the areas of aberrant CD34 expression in SECs first appear in the periportal area and then coalesce into periportal to periportal bridging and further extend to the midzonal and centrizonal areas (Fig. 3a). The areas of aberrant CD34 expression, when present, were observed in multiple areas within the examined samples and were not limited to the areas with histologically recognizable sinusoidal dilatation/congestion. Conversely, SMA expression in perisinusoidal HSCs was more frequently observed in the centrizonal and midzonal areas than in periportal areas. SMA-positive HSCs varied in size and shape but often showed stretched, long cytoplasmic processes that resulted in a confluent linear staining pattern along the perisinusoidal space of Disse (Fig. 3b). The areas showing SMA overexpression in HSCs were multifocal and not limited to the areas with histologically evident sinusoidal dilatation/congestion. SMA expression in HSCs was significantly more frequent in the SOS+ group (72.7%, 16/22) than in the control group (25%, 2/8) (*P* = 0.03), and the SOS+ group SMA score was higher (*P* = 0.02). Aberrant GS expression in the midzonal and periportal hepatocytes was less frequent compared to CD34 and SMA. All GS-positive cases were SOS+ (31.8%, 7 of 22) (Fig. 3c and d), and none of the control cases showed aberrant GS expression in either midzonal or periportal hepatocytes (0%, 0 of 8), but this difference did not reach statistical significance (*P* = 0.14). Notably, 6/7 GS-positive cases showed relatively high SOS-I (SOS-I of 3 or 4) including one case with VOD.

### Chemotherapy-induced sinusoidal injury (CSI) score

**Fig. 4:**
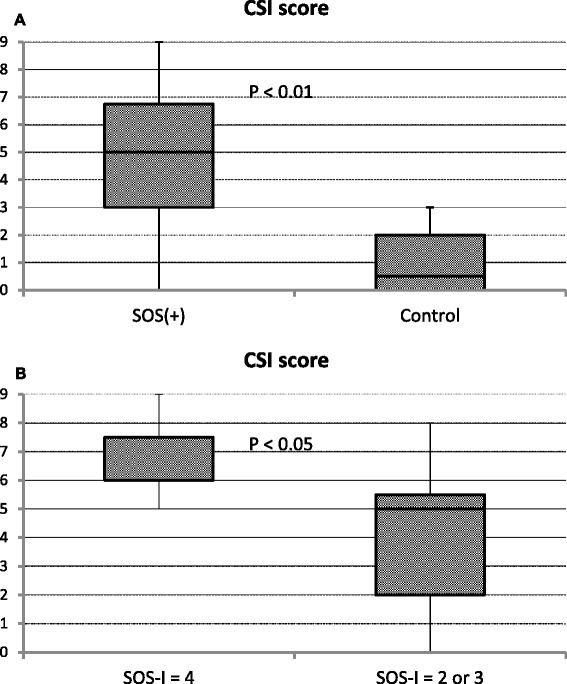
CSI score. Comparison of CSI scores between the SOS+ and control groups (**a**). Comparison of CSI score between the cases with the highest (SOS-I = 4) and lower SOS-I scores within the SOS+ group (**b**)

The CSI score was calculated as the sum of the CD34, SMA, and GS scores. The CSI score of the SOS+ group (mean ± standard deviation, 4.82 ± 2.60) was significantly higher than that of the control group (1 ± 1.20) (*P* < 0.01, Fig. 4a). Within the SOS+ group, the cases with the highest SOS-I (SOS-I = 4) showed significantly higher CSI scores (6.71 ± 1.38) than cases with lower SOS-I (SOS-I = 2 or 3) (3.93 ± 2.56) (*P* = 0.02, Fig. 4b).

## Discussion

Chemotherapy for CRLM has dramatically improved over the past 15 years, with tumor response rates increasing from 20% with 5-FU alone to 60 to 70% using variable combinations of new chemotherapeutic agents [3]. The current mainstay of chemotherapy for CRLM is FOLFIRI or FOLFOX and the standard chemotherapy backbone in first-line treatment has gradually been shifting from FOLFIRI to FOLFOX during the last decade [23, 24]. While the use of modern chemotherapy regimens has dramatically improved the CRLM response rate, its toxicity on the background liver parenchyma is becoming more recognized. OX-based regimens are associated with SEC injury, which may manifest as SOS, centrilobular, perisinusoidal, or venular fibrosis, NRH, and peliosis-like changes in severe cases [6, 25]. SOS has been associated with an increased need for intraoperative blood transfusions, an increase in the length of stay in the hospital after surgery [7], a decrease in response to chemotherapy [11, 12], early recurrence after resection and a short overall survival after resection due to liver insufficiency [26, 27].

SOS was originally termed hepatic VOD; however, recent experimental studies in pyrrolizidine alkaloid (monocrotaline)-treated rats have clarified that the main injury occurs at the level of the hepatic sinusoids, indicating that central vein involvement is not essential for SOS development [28]. SOS is caused by toxic injury to SECs and loss of sinusoidal wall integrity with subsequent sinusoid blockage by embolized sinusoidal lining cells. Impaired sinusoidal microcirculation leads to metabolic dysfunction and ischemic damage of adjacent liver cell plates, which may cause atrophy and/or dissociation of liver cell plates and further focal hepatocellular necrosis/dropout.

The histopathologic spectrum of OX-associated liver lesions was extensively investigated by Rubbia-Brandt et al. in a large series of surgically resected CRLM [9]. According to their study, 54% of patients treated with OX-based regimens had moderate/severe SOS, 47% showed centrilobular perisinusoidal/venular fibrosis, 24.5% developed NRH, and 10.6% manifested peliosis hepatis, while the 111 patients treated by surgery alone had no such lesions. Although the histologic spectrum of OX-associated liver lesions has been well documented, the precise histopathologic diagnosis of OX-associated liver injury in the setting of CRLM can be potentially difficult, due to several reasons. First, surgically resected liver specimens for CRLM contain tumor nodules of varying sizes; therefore, the histologic changes of OX-associated liver injury might be obscured by or misinterpreted as local mechanical and/or hemodynamic changes caused by tumor mass effect. This is why it is critical to sample the non-neoplastic liver away from the tumor/tumors; ideally we suggest greater than 1.5 cm away from the metastatic lesions. Second, SOS lesions can be patchy and more prominent in the subcapsular region. Uneven lesion distribution may make diagnosis difficult, particularly in biopsy samples. Even in resection specimens, it may be difficult to differentiate whether focally enhanced sinusoidal dilatation/congestion is clinically an irrelevant localized change or a true manifestation of SOS. Our stains appeared to detect SOS lesions diffusely throughout the liver parenchyma and may reduce this potential sampling error. Third, the histologic detection of perisinusoidal fibrosis and NRH changes might potentially be difficult to detect by inexperienced pathologists, especially without the use of special stains such as trichrome and reticulin, which are not routinely performed on CRLM specimens. While awareness of the histologic spectrum of OX-induced liver injury, careful examination of precise areas, and appropriate use of special stains would reduce diagnostic errors, additional markers of sinusoidal endothelial injury would further improve the diagnosis and provide a more objective assessment of this unusual type of liver injury.

Hepatic SECs are highly specialized endothelial cells characterized by fenestrations and the absence of an organized basement membrane. Fenestrae are grouped into sieve plates and control the exchange of fluids, solutes, and particles between the sinusoidal blood and the space of Disse, which contains numerous protruding microvilli of hepatocytes. Unlike vascular endothelium elsewhere in the body (except for splenic sinusoids), hepatic SECs do not express CD34 other than the small areas directly adjacent to portal tracts [29]. In normal conditions, hepatocytes and HSCs maintain the normal SEC phenotype; however, in chronic liver disease, the SECs undergo a phenotypic shift to regular vascular endothelium that is termed “sinusoidal capillarization” [30]. In such conditions, capillarized SECs lack fenestration, develop an organized basement membrane, and express CD34. Sinusoidal capillarization by means of immunohistochemistry for CD34 was observed in 81.8% of SOS+ cases, which was statistically more frequent and more extensive than in controls. To date, immunohistochemical CD34 assessment of sinusoidal capillarization in the setting of chemotherapy-induced liver injury in patients with CRLM has only been reported in a few studies. According to Narita et al., SOS was found in 39 of 80 patients treated with hepatic resection for CRLM after chemotherapy, of which 33 (85%) showed sinusoidal CD34 overexpression [14]. They also found that age over 70 years, male sex, and the presence of SOS were independent factors associated with CD34 overexpression. Nalbantoglu et al. demonstrated aberrant capillarization of the sinusoids by CD34 labeling in 98% (45/46) of cases treated with hepatic resection for CRLM after OX-based chemotherapy, and 41% (19/46) showed extensive staining throughout the lobule [15]. In their series, the overall frequency of sinusoidal capillarization by CD34 was much more frequent than that of histologically recognized OX-induced liver injury (68.1%, 32/47), and extensive multifocal CD34 expression was associated with liver injury severity. As evidenced by our study and others, sinusoidal capillarization is quite frequent in patients with CSI and CRLM and appears to be a fairly early event in the SOS disease process.

While SMA upregulation in activated HSCs has been sporadically reported in VOD of the liver after allogeneic bone marrow transplantation, SMA expression status in the setting of chemotherapy-induced sinusoidal endothelial injury in CRLM has only rarely been described [17, 18]. Sato et al. described that the area of activated HSCs significantly increased in zones 1 and 2, was more prominent in zone 3 of the hepatic lobules after bone marrow transplantation compared to normal liver tissues, and was much larger in zone 3 of liver tissues with VOD [31]. Rubbia-Brandt et al. reported strong SMA reactivity in almost all HSCs within the lobules, both along the dilated and intact sinusoids in OX-induced SOS in patients with CRLM, while sparse HSCs were SMA-positive in a small proportion of control liver lobules [18]. In our study, SMA expression in perisinusoidal HSCs was seen in 72.7% of SOS+ cases, which was more frequent and extensive than in the control group. These results are in agreement with previous descriptions and indicate that HSC activation is a salient feature of SOS.

Recent experimental studies have suggested that normal SECs of the liver function as a gatekeeper, preventing HSC activation; however, once the SEC capillarizes, it no longer prevents HSC activation and permits or promotes HSC activation and subsequent fibrosis [30]. Because drugs and toxins that cause SOS are selectively more toxic to SECs than to hepatocytes, the disease is initiated by damage at the sinusoid level. If present, prolonged loss of normal SEC with capillarization permits HSC activation and perisinusoidal fibrosis and fibrous occlusion of central venules in severe cases. Thus, given the recent experimental evidence that aberrant CD34 expression by SECs and subsequent SMA overexpression by perisinusoidal HSCs are essential in SOS initiation and development, immunohistochemical labeling for CD34 and SMA appear to be a reasonable and effective tool to identify CSI in patients with CRLM. The frequent overexpression of these markers in cases with histologically evident SOS in ours and a few other observational studies further support the feasibility of these markers as diagnostic aids in this setting.

GS is exclusively expressed in a subpopulation of hepatocytes situated adjacent to the central veins, and its peculiar zonated distribution is considered to represent a “fail-safe” mechanism for ammonia detoxification in mammals [32]. Ammonia, arriving from the intestine via the portal vein, is first metabolized by periportal hepatocytes through a low-affinity but high-capacity system involving carbamoyl phosphate synthetase and arginase 1 to generate urea. If any ammonia escapes the periportal hepatocytes, it can be scavenged and detoxified by perivenular hepatocytes through a high-affinity but low-capacity system involving GS [33]. Recently, significant expansion of the area of GS-expressing hepatocytes in human liver has been demonstrated in certain circumstances such as FNH, cirrhosis due to various causes, and idiopathic portal hypertension [19–22]. Because these conditions are well known to have altered microcirculation due to the presence of shunt vessels or abnormalities in venous and/or arterial vessels, we tested the GS expression status based on the assumption that an impaired sinusoidal microcirculation in CSI may lead to altered GS expression. In SOS, there appears to be reduced plasma access to hepatocytes due to SEC defenestration, basement membrane formation, and perisinusoidal fibrosis. In severe cases, sinusoidal perfusion might also be impaired due to microthrombi and partial or total fibrous obliteration of the central venules. Considering the fact that perivenular GS plays an important physiological role as a downstream scavenger for ammonia, it is conceivable that aberrant expression of GS in the midzonal and periportal hepatocytes in SOS may possibly represent protective recruitment of hepatocytes upstream from the perivenular area to maintain overall hepatic ammonia disposal. In our study, aberrant GS expression in the midzonal and periportal hepatocytes was observed in 31.8% of SOS+ cases. Interestingly, most GS-positive cases showed relatively high SOS-I, and none of the control cases showed aberrant GS expression, but these differences did not reach statistical significance. One limitation of our study, was a low number of control cases that had not received neoadjuvant chemotherapy prior to resection. This further supports how commonly these agents are used, and with a larger population of patients GS staining would have likely reached statistical significance.

The diagnosis of CSI should not be made based solely on the histopathologic findings. The diagnosis of CSI/SOS depends on a high index of clinical suspicion, after the clinical exclusion of other potential mimicking causes of liver injury. Several clinical features, such as hyperbilirubinemia, hepatomegaly, ascites, weight gain, and splenomegaly, have been associated with the development of SOS [34, 35], although their sensitivity and specificity have not been well defined. In the appropriate clinical setting, histological examination of the background liver of the patients with CRLM using SOS-I and the CSI scoring system would certainly increase the diagnostic accuracy of CSI. The major limitation of our study was that it compared CRLM SOS+ cases that received chemotherapy with control CRLM cases that did not. Future studies should use our panel to compare CRLM patients that received chemotherapy and are SOS positive with those that received chemotherapy and are SOS negative. This will ensure that chemotherapy use alone does not result in a positive staining pattern with our panel and an elevated CSI score. These studies, combined with correlation of CIS score to clinical features, will provide additional valuable information for the assessment of sensitivity and specificity of CIS score for clinically significant chemotherapy-related liver injury.

## Conclusions

The CSI score, calculated using an immunohistochemical panel consisting of CD34, SMA, and GS, may serve as an objective marker of chemotherapy-induced sinusoidal injury. The uniqueness of our study is although there are a few others reports using these individual markers (SMA, and CD34) as indicators of sinusoidal liver injury, ours is the first to combine them and add GS into a scoring system that can successfully diagnose CSI in liver biopsies and resection specimens in patients with CRLM. Aberrant CD34 expression by SECs and the increase in SMA-positive HSCs seem to represent the pathologic consequences of sinusoidal endothelial injury, and the altered distribution pattern of GS-expressing hepatocytes may represent an adaptive process reflecting impaired sinusoidal microcirculation. Notably, the abnormal staining patterns for CD34, SMA, and GS were not necessarily limited to the areas with sinusoidal dilatation/congestion and appear less susceptible to sampling variability in comparison with histologic evidence of sinusoidal dilatation/congestion. Therefore, the simultaneous use of these markers may increase the sensitivity for detecting CSI.

## Abbreviations

5-FU: 5-fluorouracil
CRLM: Colorectal liver metastases
CSI: Chemotherapy-induced sinusoidal injury
FNH: Focal nodular hyperplasia
GS: Glutamine synthetase
HSCs: Hepatic stellate cells
NRH: Nodular regenerative hyperplasia
OX: Oxaliplatin
SECs: Sinusoidal endothelial cells
SMA: Smooth muscle actin
SOS: Sinusoidal obstruction syndrome
SOS-I: Sinusoidal obstruction syndrome index score
VOD: Veno-occlusive disease

## References

[1] Schlag PM, Benhidjeb T, Stroszczynski C (2002). Resection and local therapy for liver metastases. Best Pract Res Clin Gastroenterol.

[2] Bartlett DL, Chu E (2012). Can metastatic colorectal cancer be cured?. Oncology (Williston Park).

[3] Folprecht G, Gruenberger T, Bechstein W, Raab HR, Weitz J, Lordick F, Hartmann JT, Stoehlmacher-Williams J, Lang H, Trarbach T, Liersch T, Ockert D, Jaeger D, Steger U, Suedhoff T, Rentsch A, Köhne CH (2014). Survival of patients with initially unresectable colorectal liver metastases treated with FOLFOX/cetuximab or FOLFIRI/cetuximab in a multidisciplinary concept (CELIM study). Ann Oncol.

[4] McWhirter D, Kitteringham N, Jones RP, Malik H, Park K, Palmer D (2013). Chemotherapy induced hepatotoxicity in metastatic colorectal cancer: a review of mechanisms and outcomes. Crit Rev Oncol Hematol.

[5] Nguyen-Khac E, Lobry C, Chatelain D, Fuks D, Joly JP, Brevet M, Tramier B, Mouly C, Hautefeuille V, Chauffert B, Regimbeau JM. A reappraisal of chemotherapy-induced liver injury in colorectal liver metastases before the era of antiangiogenics. Int J Hepatol. 2013;2013(314868):11. 10.1155/2013/314868 PMC3606725 23533786

[6] Robinson SM, Wilson CH, Burt AD, Manas DM, White SA (2012). Chemotherapy-associated liver injury in patients with colorectal liver metastases: a systematic review and meta-analysis. Ann Surg Oncol.

[7] Kneuertz PJ, Maithel SK, Staley CA, Kooby DA (2011). Chemotherapy-associated liver injury: impact on surgical management of colorectal cancer liver metastases. Ann Surg Oncol.

[8] Fan CQ, Crawford JM (2014). Sinusoidal obstruction syndrome (hepatic veno-occlusive disease). J Clin Exp Hepatol.

[9] Rubbia-Brandt L, Lauwers GY, Wang H, Majno PE, Tanabe K, Zhu AX, Brezault C, Soubrane O, Abdalla EK, Vauthey JN, Mentha G, Terris B (2010). Sinusoidal obstruction syndrome and nodular regenerative hyperplasia are frequent oxaliplatin-associated liver lesions and partially prevented by bevacizumab in patients with hepatic colorectal metastasis. Histopathology.

[10] Morine Y, Shimada M, Utsunomiya T (2014). Evaluation and management of hepatic injury induced by oxaliplatin-based chemotherapy in patients with hepatic resection for colorectal liver metastasis. Hepatol Res.

[11] Vreuls CP, Van Den Broek MA, Winstanley A, Koek GH, Wisse E, Dejong CH, Olde Damink SW, Bosman FT, Driessen A (2012). Hepatic sinusoidal obstruction syndrome (SOS) reduces the effect of oxaliplatin in colorectal liver metastases. Histopathology.

[12] Viganò L, Capussotti L, De Rosa G, De Saussure WO, Mentha G, Rubbia-Brandt L (2013). Liver resection for colorectal metastases after chemotherapy: impact of chemotherapy-related liver injuries, pathological tumor response, and micrometastases on long-term survival. Ann Surg.

[13] Narita M, Oussoultzoglou E, Chenard MP, Rosso E, Casnedi S, Pessaux P, Bachellier P, Jaeck D (2011). Sinusoidal obstruction syndrome compromises liver regeneration in patients undergoing two-stage hepatectomy with portal vein embolization. Surg Today.

[14] Narita M, Oussoultzoglou E, Chenard MP, Fuchshuber P, Rather M, Rosso E, Addeo P, Jaeck D, Bachellier P (2012). Liver injury due to chemotherapy-induced sinusoidal obstruction syndrome is associated with sinusoidal capillarization. Ann Surg Oncol.

[15] Nalbantoglu IL, Tan BR, Linehan DC, Gao F, Brunt EM (2014). Histological features and severity of oxaliplatin-induced liver injury and clinical associations. J Dig Dis.

[16] Blomhoff R, Wake K (1991). Perisinusoidal stellate cells of the liver: important roles in retinol metabolism and fibrosis. FASEB J.

[17] Kang GH, Moon HS, Lee ES, Kim SH, Sung JK, Lee BS, Jeong HY, Lee HY, Kang DY (2013). A case of liver fibrosis with splenomegaly after oxaliplatin-based adjuvant chemotherapy for colon cancer. J Korean Med Sci.

[18] Rubbia-Brandt L, Audard V, Sartoretti P, Roth AD, Brezault C, Le Charpentier M, Dousset B, Morel P, Soubrane O, Chaussade S, Mentha G, Terris B (2004). Severe hepatic sinusoidal obstruction associated with oxaliplatin-based chemotherapy in patients with metastatic colorectal cancer. Ann Oncol.

[19] Bioulac-Sage P, Laumonier H, Rullier A, Cubel G, Laurent C, Zucman-Rossi J, Balabaud C (2009). Over-expression of glutamine synthetase in focal nodular hyperplasia: a novel easy diagnostic tool in surgical pathology. Liver Int.

[20] Joseph NM, Ferrell LD, Jain D, Torbenson MS, Wu TT, Yeh MM, Kakar S (2014). Diagnostic utility and limitations of glutamine synthetase and serum amyloid-associated protein immunohistochemistry in the distinction of focal nodular hyperplasia and inflammatory hepatocellular adenoma. Mod Pathol.

[21] Fleming KE, Wanless IR (2013). Glutamine synthetase expression in activated hepatocyte progenitor cells and loss of hepatocellular expression in congestion and cirrhosis. Liver Int.

[22] Sato Y, Harada K, Sasaki M, Nakanuma Y (2015). Altered intrahepatic microcirculation of idiopathic portal hypertension in relation to glutamine synthetase expression. Hepatol Res.

[23] Song X, Zhao Z, Barber B, Gregory C, Wang PF, Long SR (2011). Treatment patterns and metastasectomy among mCRC patients receiving chemotherapy and biologics. Curr Med Res Opin.

[24] Hess GP, Wang PF, Quach D, Barber B, Zhao Z (2010). Systemic therapy for metastatic colorectal cancer: patterns of chemotherapy and biologic therapy use in US medical oncology practice. J Oncol Pract.

[25] Schwarz RE, Berlin JD, Lenz HJ, Nordlinger B, Rubbia-Brandt L, Choti MA (2013). Systemic cytotoxic and biological therapies of colorectal liver metastases: expert consensus statement. HPB (Oxford).

[26] Nakano H, Oussoultzoglou E, Rosso E, Casnedi S, Chenard-Neu MP, Dufour P, Bachellier P, Jaeck D (2008). Sinusoidal injury increases morbidity after major hepatectomy in patients with colorectal liver metastases receiving preoperative chemotherapy. Ann Surg.

[27] Tamandl D, Klinger M, Eipeldauer S, Herberger B, Kaczirek K, Gruenberger B, Gruenberger T (2011). Sinusoidal obstruction syndrome impairs long-term outcome of colorectal liver metastases treated with resection after neoadjuvant chemotherapy. Ann Surg Oncol.

[28] DeLeve LD, Shulman HM, McDonald GB (2002). Toxic injury to hepatic sinusoids: sinusoidal obstruction syndrome (veno-occlusive disease). Semin Liver Dis.

[29] Pusztaszeri MP, Seelentag W, Bosman FT (2006). Immunohistochemical expression of endothelial markers CD31, CD34, von Willebrand factor, and Fli-1 in normal human tissues. J Histochem Cytochem.

[30] DeLeve LD (2015). Liver sinusoidal endothelial cells in hepatic fibrosis. Hepatology.

[31] Sato Y, Asada Y, Hara S, Marutsuka K, Tamura K, Hayashi T, Sumiyoshi A (1999). Hepatic stellate cells (Ito cells) in veno-occlusive disease of the liver after allogeneic bone marrow transplantation. Histopathology.

[32] Gebhardt R, Baldysiak-Figiel A, Krügel V, Ueberham E, Gaunitz F (2007). Hepatocellular expression of glutamine synthetase: an indicator of morphogen actions as master regulators of zonation in adult liver. Prog Histochem Cytochem.

[33] Colnot S, Perret C, Monga SPS (2011). Liver zonation. Molecular pathology of liver diseases.

[34] Dignan FL, Wynn RF, Hadzic N, Karani J, Quaglia A, Pagliuca A, Veys P, Potter MN, Haemato-oncology Task Force of British Committee for Standards in Haematology; British Society for Blood and Marrow Transplantation (2013). BCSH/BSBMT guideline: diagnosis and management of veno-occlusive disease (sinusoidal obstruction syndrome) following haematopoietic stem cell transplantation. Br J Haematol.

[35] Overman MJ, Maru DM, Charnsangavej C, Loyer EM, Wang H, Pathak P, Eng C, Hoff PM, Vauthey JN, Wolff RA, Kopetz S (2010). Oxaliplatin-mediated increase in spleen size as a biomarker for the development of hepatic sinusoidal injury. J Clin Oncol.

